# Supplementary material for: Bclaf1 critically regulates the type I interferon response and is degraded by alphaherpesvirus US3
Source: PLoS Pathog. 2019 Jan 25;15(1):e1007559. doi: 10.1371/journal.ppat.1007559 (PMC6364948; doi:10.1371/journal.ppat.1007559)
Supplement: S1 Table — (DOCX) [file ppat.1007559.s001.docx]

| **Primer Name**  **S1 Table: Primers used in this study.** | **Sequence (5'-3')** | **Purpose** |
| --- | --- | --- |
| h*GAPDH-*F | AGCCAAAAGGGTCATCATCTC | qPCR primers for human *GAPDH* |
| h*GAPDH-*R | GGACTGTGGTCATGAGTCCTTC |  |
| h*ISG15-*F | CAGATCACCCAGAAGATCG | qPCR primers for human *ISG15* |
| h*ISG15-*R | CCCTTGTTATTCCTCACCAG |  |
| h*IFIT1*-F | ACACCTGAAAGGCCAGAATGAGGA | qPCR primers for human *IFIT1* |
| h*IFIT1*-R | TGCCAGTCTGCCCATGTGGTAATA |  |
| h*IFIT2*-F | GACACGGTTAAAGTGTGGAG | qPCR primers for human *IFIT2* |
| h*IFIT2*-R | GGTACTGGTTGTCAGGATTC |  |
| h*OAS1*-F | CTACCCTGTGTGTGTGTCCAAG | qPCR primers for human *OAS1* |
| h*OAS1*-R | CACGGAAAATGCTCTCTCTCTT |  |
| p*GAPDH-*F | ACATGGCCTCCAAGGAGTAAGA | qPCR primers for porcine *GAPDH* |
| p*GAPDH-*R | GATCGAGTTGGGGCTGTGACT |  |
| p*ISG15-*F | GGTGCAAAGCTTCAGAGACC | qPCR primers for porcine *ISG15* |
| p*ISG15-*R | GTCAGCCAGACCTCATAGGC |  |
| p*IFIT1*-F | TCAGAGGTGAGAAGGCTGGT | qPCR primers for porcine *IFIT1* |
| p*IFIT1*-R | GCTTCCTGCAAGTGTCCTTC |  |
| p*MX1*-F | AGCGCAGTGACACCAGCGAC | qPCR primers for porcine *MX1* |
| p*MX1*-R | GCCCGGTTCAGCCTGGGAAC |  |
| *Bclaf1*-F | TCACCTGAGCAGGTAAAGTCTGA | qPCR primers for human or porcine *Bclaf1* |
| *Bclaf1*-R | AGTCAAGGAAGCAGGTCTGTTAG |  |
| *IE180*-F | CATCGTGCTGGACACCATCGAG | qPCR primers for PRV *IE180* |
| *IE180*-R | ACGTAGACGTGGTAGTCCCCC A |  |
| *ICP4*-F | GACGTGCGCGTGGTGGTGCTGTACTCG | qPCR primers for HSV-1 *ICP4* |
| *ICP4*-R | GCGCACGGTGTTGACCACGATGAGCC |  |
| *ISG15*(ChIP)*-*F | CGCCACTTTTGCTTTTCCCT | PCR primers for human *ISG15* in ChIP assays |
| *ISG15*(ChIP)*-*R | ATAAGCCTGAGGCACACACG |  |
| *IFIT1*(ChIP)-F | TTGGGTTTCTGCAGCACTAGA | PCR primers for human *IFIT1* in ChIP assays |
| *IFIT1*(ChIP)-R | ACCTAGGGAAACCGAAAGGG |  |
| *IFIT2*(ChIP)-F | CTTTCCCTTTTGTAACGTCAGC | PCR primers for human *IFIT2* in ChIP assays |
| *IFIT2*(ChIP)-R | TGCACTCTTCAGAAATCTTCCTC |  |
| *IFIT1 Exon2*(ChIP)-F | CTCTGCCTATCGCCTGGATG | PCR primers for human *IFIT1 Exon2* in ChIP assays |
| *IFIT1 Exon2*(ChIP)-R | CCTGCCTTAGGGGAAGCAAA |  |
| Bclaf1 exon4-F | ACCGAGAGGAATGAGACGACCTTA | Encoding sgRNA for Cas9 targeting exon 4 of Bclaf1 to generate Bclaf1-KO HeLa cells |
| Bclaf1 exon4-R | AAACTAAGGTCGTCTCATTCCTCT |  |
| Bclaf1 intron-F | ACCGATCGACAGTACCTGTCATGC | Encoding sgRNA for Cas9 targeting intron of Bclaf1 to generate HEp-2^Flag-Bclaf1^ cells |
| Bclaf1 intron -R | AAACGCATGACAGGTACTGTCGAT |  |

| PRV US3 Target1-F | ACCGCTCGTCGGGGATTCCGGCGT | Encoding sgRNA for Cas9 targeting US3 gene to generate PRV ∆US3 virus |
| --- | --- | --- |
| PRV US3 Target1-R | AAACACGCCGGAATCCCCGACGAG |  |
| PRV US3 Target2-F | ACCGCGTCTTCGTGGCCCGCCGGC |  |
| PRV US3 Target2-R | AAACGCCGGCGGGCCACGAAGACG |  |
| HSV-1 US3 Targer-F | ACCGAGTCCTGGTTTCCGTACATC | Encoding sgRNA for Cas9 targeting US3 gene to generate HSV-1 ∆US3 virus |
| HSV-1 US3 Targer-R | AAACGATGTACGGAAACCAGGACT |  |
