# Supplementary figures and images for: Bclaf1 critically regulates the type I interferon response and is degraded by alphaherpesvirus US3
Source: PLoS Pathog. 2019 Jan 25;15(1):e1007559. doi: 10.1371/journal.ppat.1007559 (PMC6364948; doi:10.1371/journal.ppat.1007559)

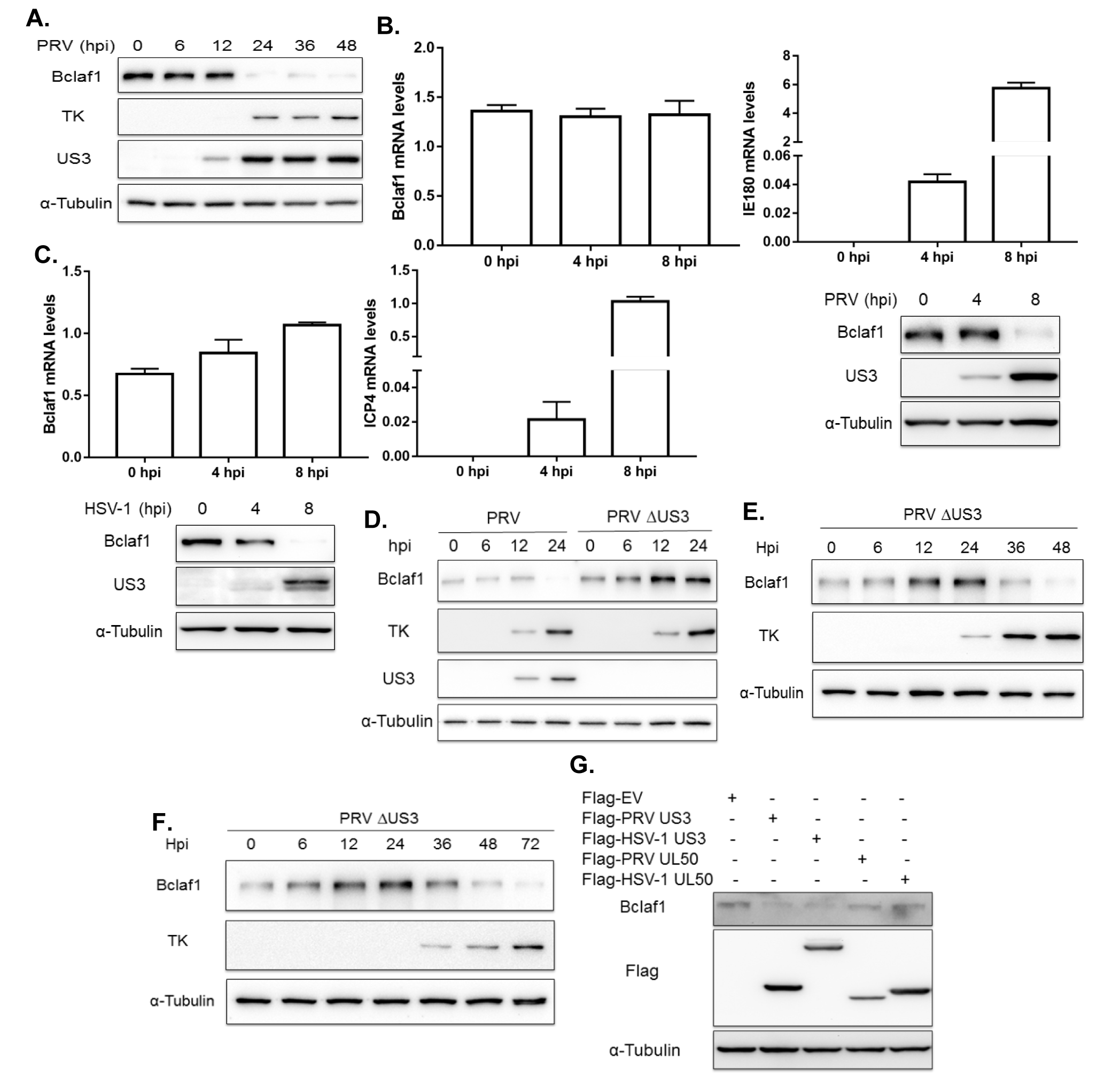

Supplement: S1 Fig — (A) IB analysis of Bclaf1, TK and US3 in ST cells infected with PRV (MOI = 1) for the indicated hours. (B) qRT-PCR analysis of Bclaf1 and IE180 mRNA levels and IB analysis of Bclaf1 and US3 in PK15 cells infected with PRV for the indicated hours. (C) qRT-PCR analysis of Bclaf1 and ICP4 mRNA levels and IB analysis of Bclaf1 and US3 in HEp-2 cells infected with HSV-1 for the indicated hours. (D) IB analysis of Bclaf1, TK and US3 in ST cells infected with PRV WT or PRV ΔUS3 (MOI = 1) for the indicated hours. (E and F) IB analysis of Bclaf1 and TK in ST cells infected with PRV ΔUS3 at 1 moi(E) or 0.1 moi(F) for the indicated hours. (G) IB analysis of endogenous Bclaf1 in HEK293T cells transfected with Flag-tagged PRV US3, HSV-1 US3, PRV UL50 or HSV-1 UL50 expression plasmids. (TIF) [file ppat.1007559.s002.tif]

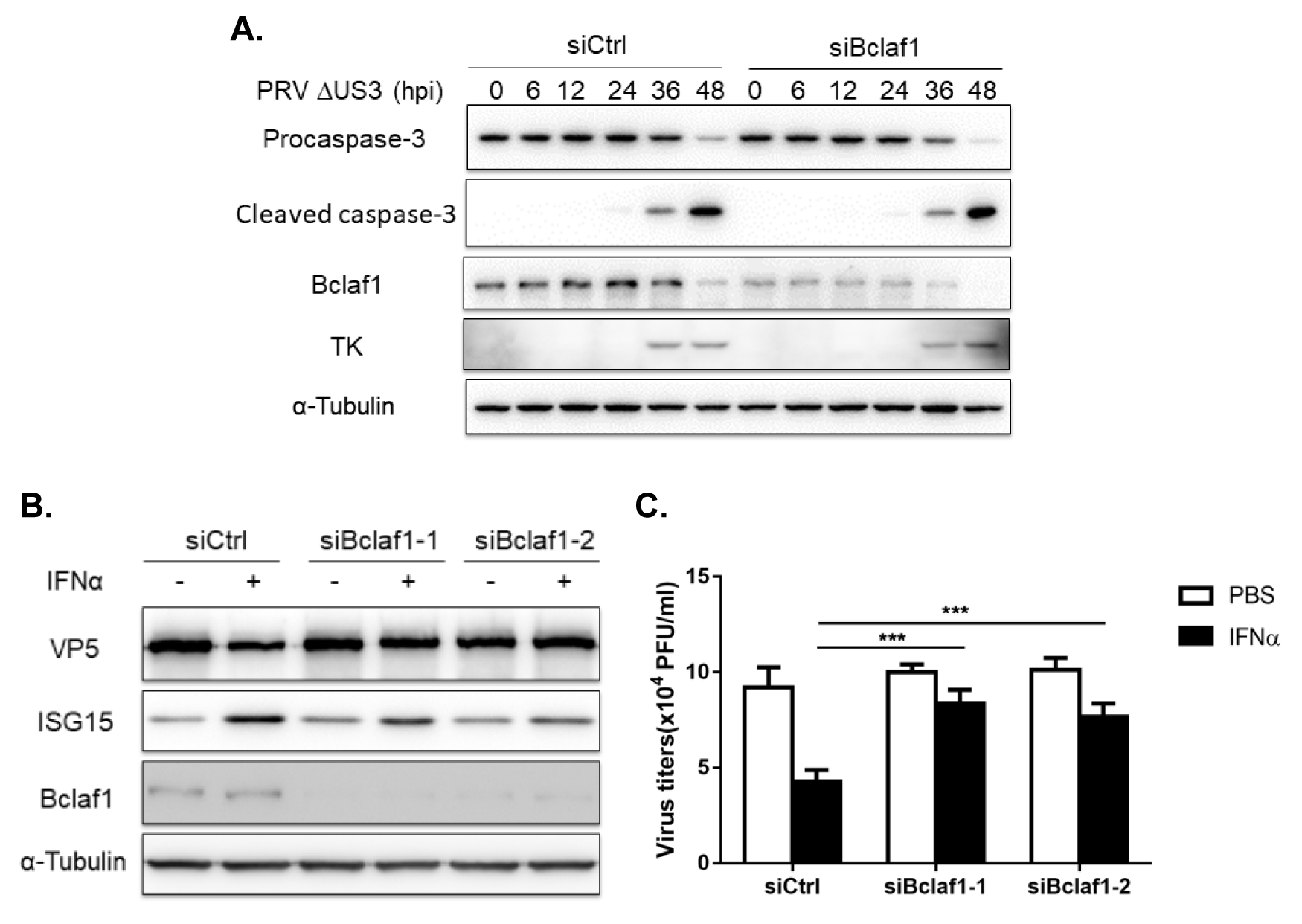

Supplement: S2 Fig — (A) IB analysis of caspase3, Bclaf1 and TK in ST cells infected with PRV ΔUS3 (MOI = 0.1) for the indicated hours. (B) IB analysis of VP5, ISG15 and Bclaf1 in HEp-2 cells transfected with si-control or si-Bclaf1 followed by PBS or human IFNα (500U/mL) treatment for 12h and then infected with HSV-1 ΔUS3 (MOI = 3) for 24h. (C) Plaque assay analyzed titers of virus in supernatants as described in (B). Data are shown as mean ± SD of three independent experiments. Statistical analysis was performed by the two-way ANOVA test. ***p<0.001. (TIF) [file ppat.1007559.s003.tif]

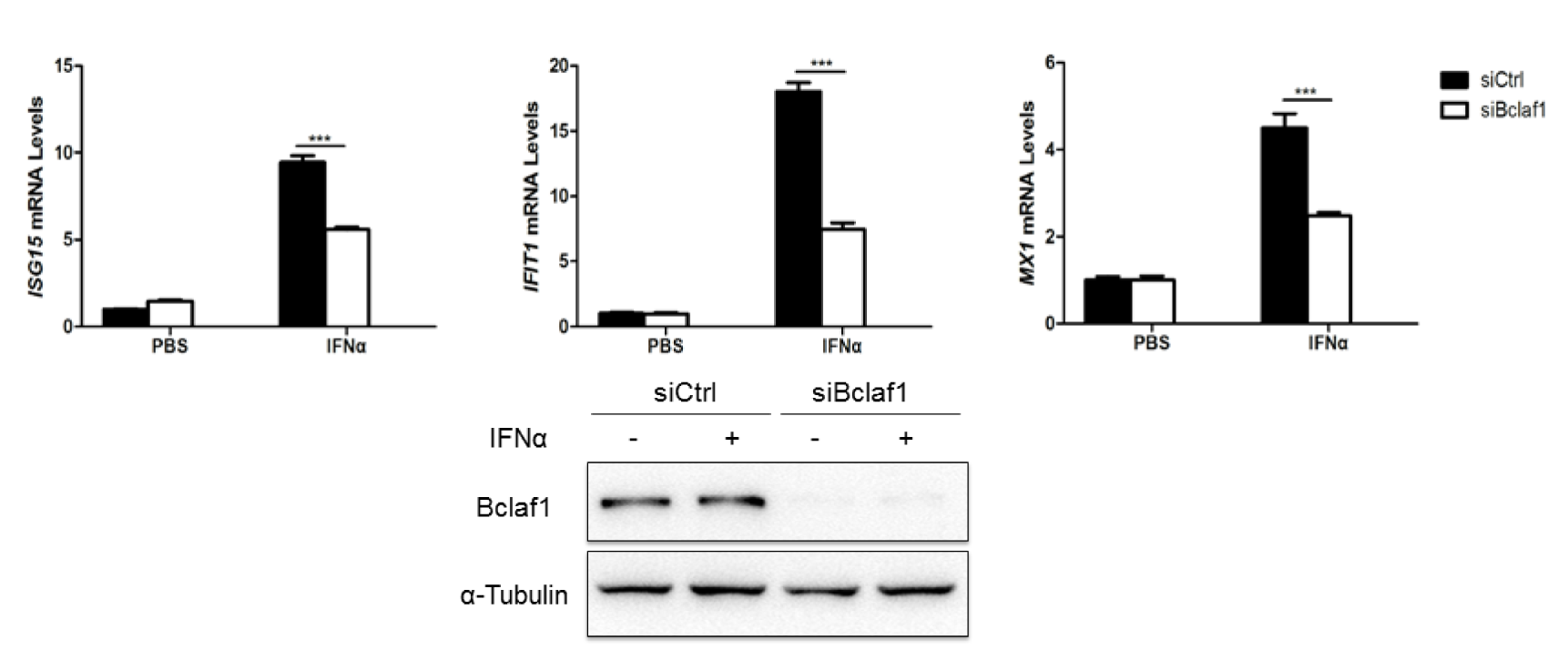

Supplement: S3 Fig — qRT-PCR analysis of ISG15, IFIT1 and MX1 mRNA levels in PK15 cells transfected with si-control or si-Bclaf1 followed by PBS or porcine IFNα (500U/mL) treatment for 2h. IB analyzed the knocking down efficiency. Data are shown as mean ± SD of three independent experiments. Statistical analysis was performed by the two-way ANOVA test (A, C and D). ***p<0.001. (TIF) [file ppat.1007559.s004.tif]

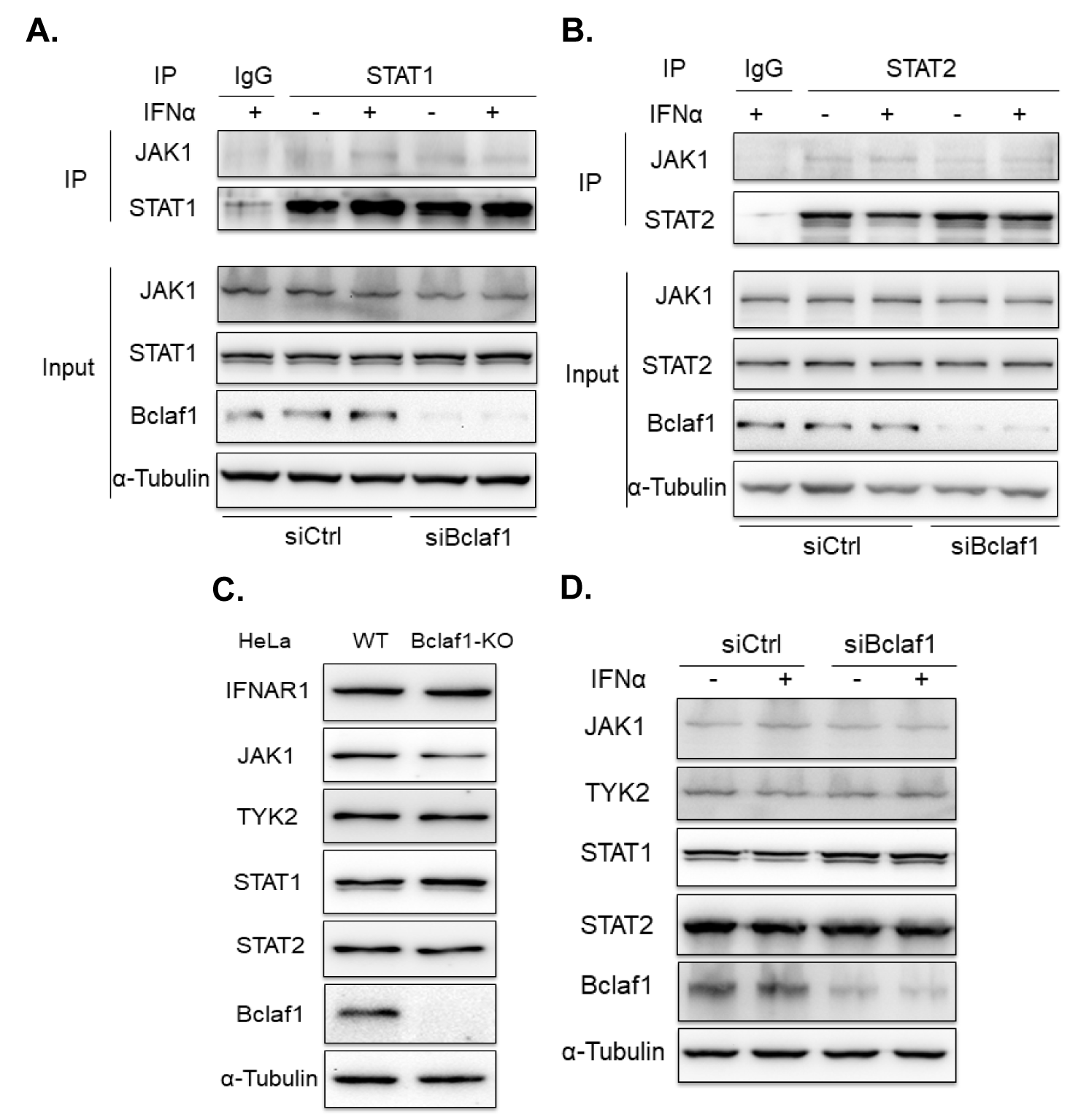

Supplement: S4 Fig — (A and B) IB analysis of JAK1, STAT1 or STAT2 in immunoprecipitates and whole-cell lysates of HEp-2 cells transfected with si-control or si-Bclaf1 followed by PBS or human IFNα (500U/mL) treatment for 30 min. (C) IB analysis of IFNAR1, JAK1, TYK2, STAT1, STAT2 and Bclaf1 in HeLa WT and HeLa Bclaf1-KO cells. (D) IB analysis of JAK1, TYK2, STAT1, STAT2 and Bclaf1 in HEp-2 cells transfected with si-control or si-Bclaf1 followed by PBS or human IFNα (500U/mL) treatment for 1h. (TIF) [file ppat.1007559.s005.tif]

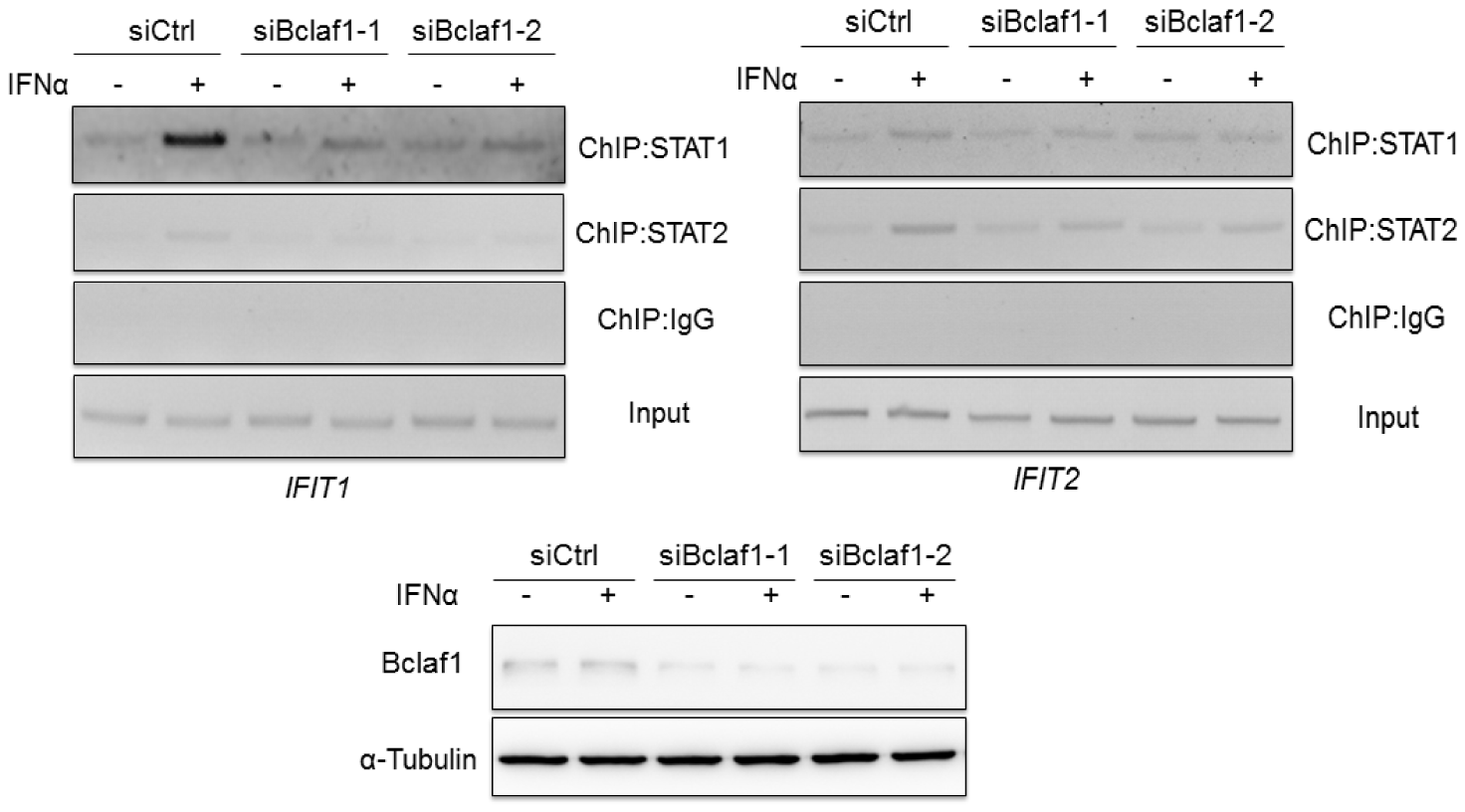

Supplement: S5 Fig — ChIP analysis of STAT1/STAT2 DNA-binding in promoters of IFIT1 and IFIT2 in HEp-2 cells transfected with si-control or si-Bclaf1 followed by PBS or human IFNα (500U/mL) treatment for 1h. IB analyzed the knocking down efficiency. (TIF) [file ppat.1007559.s006.tif]

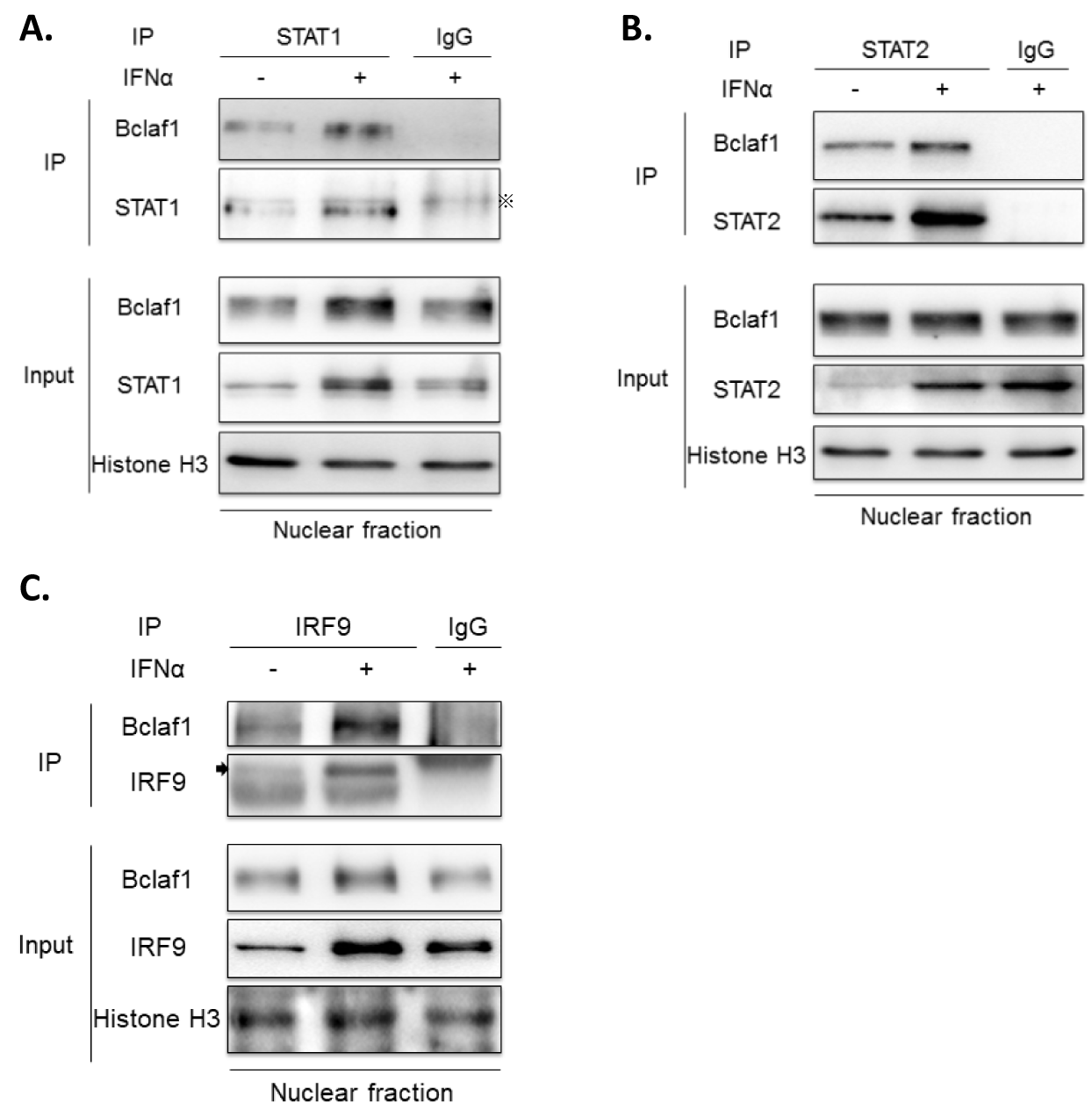

Supplement: S6 Fig — (A) IB analysis of Bclaf1 and STAT1 in nuclear immunoprecipitates of HeLa cells treated with PBS or human IFNα (500U/mL) for 2h. The asterisk(※) indicated a nonspecific band from IgG. (B) IB analysis of Bclaf1 and STAT2 in nuclear immunoprecipitates of HeLa cells treated with PBS or human IFNα (500U/mL) for 2h. (C) IB analysis of Bclaf1 and IRF9 in nuclear immunoprecipitates of HeLa cells treated with PBS or human IFNα (500U/mL) for 4h. The arrows indicated the bands of IRF9. (TIF) [file ppat.1007559.s007.tif]
